# Explicit and Hybrid Solvent Models for Estimates of Parameters Relevant to the Reduction Potential of Ethylene Carbonate

**DOI:** 10.3390/ijms232415590

**Published:** 2022-12-09

**Authors:** Andrzej Eilmes, Piotr Kubisiak, Piotr Wróbel

**Affiliations:** Faculty of Chemistry, Jagiellonian University, Gronostajowa 2, 30-387 Kraków, Poland

**Keywords:** ethylene carbonate, reduction potential, solvent model, quantum-chemical calculations, frontier orbitals

## Abstract

Using ethylene carbonate as a sample solvent, we investigated two molecular parameters used to estimate the reduction potential of the solvent: electron affinity, and the energy of the lowest unoccupied molecular orbital (LUMO). The results showed that the values of these parameters are inconsistent for a single ethylene carbonate molecule in vacuum calculations and in the continuous effective solvent. We performed a series of calculations employing explicit or hybrid (explicit/continuous) solvent models for aggregates of solvent molecules or solvated salt ions. In the hybrid solvent model, values of the two estimates extrapolated to an infinite system size converged to one common value, whereas the difference of 1 eV was calculated in the purely explicit solvent. The values of the gap between the highest occupied molecular orbital (HOMO) and the LUMO obtained in the hybrid model were significantly larger than those resulting from the explicit solvent calculations. We related these differences to the differences in frontier orbitals and changes of electron density obtained in the two solvent models. In the hybrid solvent model, the location of the additional electron in the reduced system usually corresponds to the LUMO orbital of the oxidized system. The presence of salt ions in the solvent affects the extrapolated values of the electron affinity and LUMO energy.

## 1. Introduction

With the challenges of the sustainable economy stimulating an increasing demand for cheap, stable, environmentally friendly and safe energy storage devices, a large effort is being invested in research on rechargeable batteries [1]. A key component of a metal-ion battery is the ion-conducting electrolyte, and its properties play an essential role for safety of use and the stability of the device.

The reduction and oxidation potentials of the electrolyte determine its electrochemical stability under working conditions. It is therefore unsurprising that, in addition to experimental research, methods of computational modeling are used to calculate the redox potentials. Several recent works investigated the performance of quantum chemical calculations for the prediction of reduction and oxidation potentials of a number of solvents [2,3,4], including ethylene carbonate (EC), commonly used in lithium-ion batteries. The reduction mechanisms of carbonates are intensively studied because of the crucial role of electrolyte decomposition in the formation of a solid-electrolyte interphase layer [2,5,6,7,8,9].

Reduction potential *E_r_* for a one-electron reaction:

O + *e*^−^ → R, (1)

where O and R are an oxidized and a reduced reagent, respectively, can be computed from the formula

(2) $$E_{r}=-\frac{\Delta G}{F}$$

where Δ*G* is the change of the Gibbs free energy associated with the process 1 and *F* is the Faraday constant. The Δ*G* value in the solvent must account for solvation effects and therefore it is usually calculated using a thermochemical cycle [10]. Nevertheless, the thermal corrections to *G* are small with respect to the estimated potential, and therefore the approximate reduction potential can be obtained from the difference of electronic energies of the system, with the solvation effects included e.g., via a continuous solvent model [3]:

(3) $$E_{r}=-\frac{E_{red}-E_{ox}}{F}$$

where *E_red_* and *E_ox_* are the electronic energies of the system in the reduced and the oxidized state. For a one-electron process, the system in the reduced state contains one electron more than in the initial oxidized state and therefore

(4) $$E_{r}=\frac{\mathrm{EA}}{F}$$

with EA being the electron affinity of the system in its oxidized state. The EA value may be approximated from the LUMO energy in the oxidized reagent:

−EA = *E*_LUMO_, (5)

hence

(6) $$E_{r}=-\frac{E_{\mathrm{LUMO}}}{F}$$

Applying these relations to ethylene carbonate molecule one can note that formulas 4 and 5 yield quite different results. In the work [2] values −EA = 1.06 eV and *E*_LUMO_ = −0.38 eV were obtained for an EC molecule in vacuum. In the effective solvent with the dielectric constant corresponding to water these values changed to −EA = −0.65 eV and *E*_LUMO_ = 0.06 eV, but there is still a difference of about 0.7 eV.

The *E*_LUMO_ value is often used to estimate the reduction potentials, partly because it is simple to obtain without computationally demanding calculations. The difference between −EA and *E*_LUMO_ values is therefore a disadvantageous feature. The estimates discussed above were calculated for a single solvent molecule, in vacuum or in a continuous solvent model. We asked, therefore, the question of whether the discrepancy between −EA and *E*_LUMO_ may be attributed to the description of the solvent effects and what the difference might be between these two values when an explicit solvent model is used, so the system under investigation contains many solvent molecules.

The explicit solvent calculations can be applied to estimate the solvation contributions to the free energy based on molecular dynamics (MD) simulations [10], as in recent works [11,12]. We did not attempt such an advanced modeling in our study, focusing instead on a simple question whether the increase in the explicit part of the solvent in the explicit or hybrid solvent models can drive −EA and *E*_LUMO_ toward convergence to a common value, which may be then used in the estimate of the reduction potential. Although some studies investigating systems with few EC molecules are reported in the literature, they were more focused on the Li^+^-solvent complexes, rather than on the solvent itself and used only selected system sizes [13]. Our work was aimed at more systematic investigations concerning how the solvent parameters evolve with the increasing number of solvent molecules.

Our model system was ethylene carbonate as one of solvents used in commercial batteries [14]. The important property of an EC-based electrolyte is the formation of an effective protective layer on a graphitic anode in a reduction process of EC molecules [15]. In addition to neat liquid, we also investigated the electrolyte with dissolved LiTFSI salt (TFSI = bis(trifluoromethanesulfonyl)imide). LiTFSI is studied as an alternative salt for Li-ion devices [14], as a solution in carbonate solvents [16] or in a mixture with an ionic liquid [17].

## 2. Results

Based on the optimized geometries and frequencies we calculated Gibbs free energy change in the reduction of a single EC molecule, EA, and the LUMO energy for EC. The results are collected in Table 1.

Indeed, the difference between ΔG and EA values was relatively small, justifying the approximation introduced in equation 3. In vacuum there was a difference of about 0.9 eV between −EA and LUMO energy; the disagreement between these two estimates became even larger (1.3–1.4 eV) in the polarizable continuum model (PCM) calculations. These results confirmed our observation from the literature data stated in Section 1.

**Figure 1:**
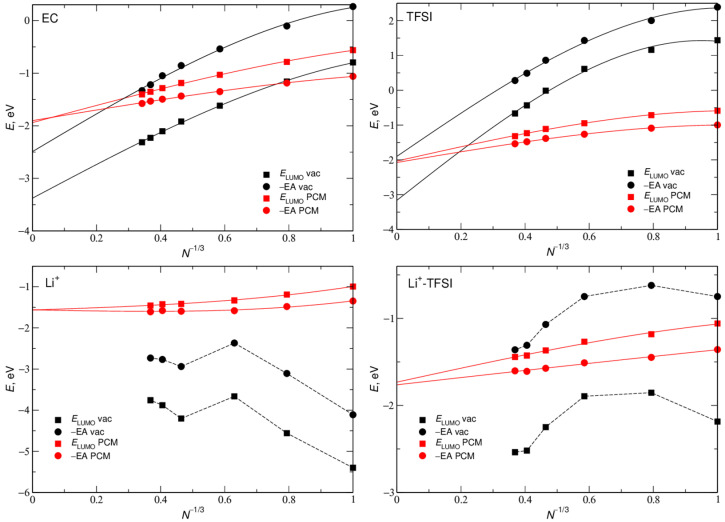
Electron affinity and LUMO energy calculated in vacuum and in the PCM for aggregates containing *N* solvent molecules with the core of the aggregate being EC molecule, ion or ion pair. Solid lines are fits to the data.

Therefore, we turned our attention to the dependences observed in the explicit and in the hybrid solvent model. In Figure 1 we show the averaged values of −EA and *E*_LUMO_ plotted vs. the *N*^−1/3^. To obtain the values corresponding to an infinite *N* (bulk liquid solvation), the results were extrapolated to *N*^−1^ → 0. We used the formula

*E* = *a*_0_ + *a*_1_*N*^−1^ + *a*_2_*N*^−1/3^, (7)

to fit the energies, assuming that the energy depends on the number of EC molecules in the system *N* and on the radius of the cluster, proportional to *N*^1/3^. The *a*_0_ parameter of the fit gives the estimate of the energy extrapolated to an infinite system size. The data for solvated Li^+^ and LiTFSI obtained from vacuum calculations change with *N* non-monotonically; this behavior is related to the increased stability of Li^+^(EC)_4_ cluster. Therefore, fitting was not performed for these results. The energies resulting from extrapolations are collected in Table 2.

The LUMO energies and the −EA values for neutral or negatively charged systems generally decreased with the system size. The exceptions were the positively charged clusters with the Li^+^ core for which the changes in the PCM calculations were small and there was an increase of energies observed in the vacuum results.

It may be noted that for all kinds of studied systems, in the vacuum calculations, the *E*_LUMO_ energy is lower than the value of −EA; the difference exceeds 1 eV and does not change significantly with the system size. The opposite was observed in the PCM results: the *E*_LUMO_ was higher than −EA, the difference between the two values was smaller than 0.5 eV and decreases with increasing *N*.

**Figure 2:**
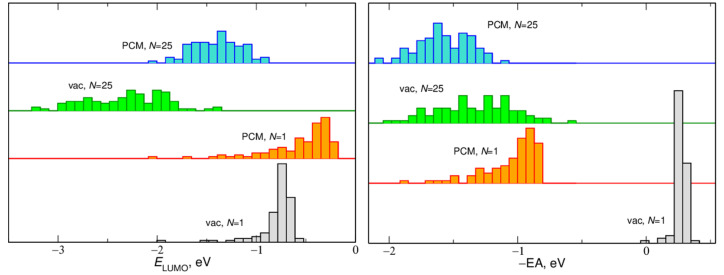
Distributions of EA and *E*_LUMO_ values calculated for aggregates of *N* EC molecules.

**Figure 3:**
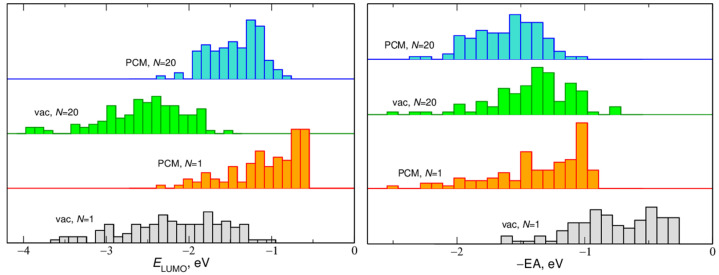
Distributions of EA and *E*_LUMO_ values calculated for LiTFSI ion pair solvated in *N* EC molecules.

In addition to the average values, we also checked the distributions of −EA and *E*_LUMO_ energies. Sample results are presented in Figure 2 for EC clusters and in Figure 3 for solvated LiTFSI ion pair. The width of the histograms was the smallest for a single EC molecule in vacuum. In this case, different energy values resulted from different geometries of the molecule extracted from different frames of the MD trajectory. Distributions of −EA and *E*_LUMO_ values calculated for one EC molecule in PCM were much wider and asymmetric—values closer to 0 are more probable. The width of distributions calculated for 25 EC molecules both in vacuum and in the PCM was in the range 1–1.5 eV and the distributions were approximately symmetric. The histograms for *N* = 25 obtained in the PCM calculations were noticeably narrower than those corresponding to the vacuum results. Similar trends were observed for the LiTFSI pair solvated in EC molecules, with the notable exception that for *N* = 1 the distributions were very wide; the effect originated from different configurations of Li^+^ and TFSI ions and the solvent molecule.

Another parameter of the molecular system relevant to the electrochemical stability of the electrolyte is the HOMO–LUMO gap

**Figure 4:**
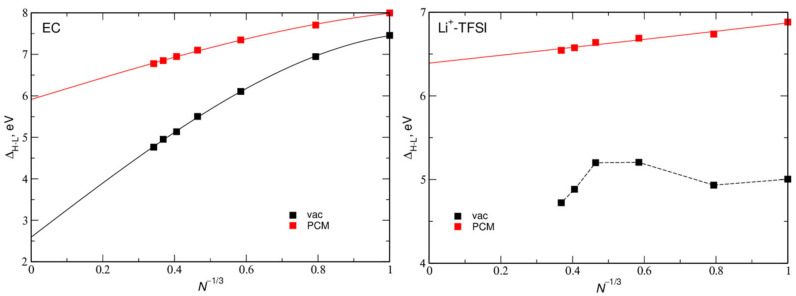
HOMO–LUMO gap calculated for aggregates of *N* EC molecules and for LiTFSI ion pair solvated in *N* EC molecules. Solid lines are fits to the data.

Δ_H-L_ = *E*_LUMO_ − *E*_HOMO_, (8)

providing an estimate of the electrochemical window. In Figure 4 we present the Δ_H-L_ values for EC aggregates and solvated LiTFSI, with the fits according to the Equation (7). The values extrapolated to the infinite system size are collected in Table 3. The gap values decreased with *N* and the values obtained in vacuum were about 2–2.5 eV lower than those calculated in the implicit PCM solvent. The largest Δ_H-L_ value was obtained for solvated ion pair.

## 3. Discussion

It is readily seen, from the data shown in Figure 1, that the two approaches to the solvent modeling used in our calculations are inequivalent and the results from the explicit and the hybrid model do not converge to one common value in the limit of infinite system size.

As expected from the calculations for a single EC molecule, there is a significant difference between −EA and *E*_LUMO_ values for EC *N* = 1 data. The values given in Table 1 differ from the averages shown in Figure 1, because the former were obtained for geometries optimized in density functional theory (DFT) calculations both for neutral molecule and for the anion, whereas the latter energies were calculated at the geometries extracted from the MD structures. In the PCM calculations *E*_LUMO_ is 1.3 eV higher than the −EA value.

For all studied systems, in PCM calculations the difference between electron affinity and LUMO energy decreases with *N*, so that both energies converge to the same value. The extrapolated values (Table 2) differ less than by 0.05 eV, therefore our working hypothesis has been confirmed. Comparing the data for EC clusters and solvated ions we noted that the presence of Li^+^ cation increased the energy whereas the TFSI anion decreased the energies. Therefore the −EA and *E*_LUMO_ for EC clusters (~−1.9 eV) are between the values obtained for Li^+^ in EC (~−1.6 eV) and for TFSI in EC (~−2 eV). These differences seem to be related to the electrostatic stabilization of the system in the polarizable medium: for EC and LiTFSI in EC the oxidized system is neutral, and the reduced system is charged, whereas the opposite is true for Li^+^ in EC. For TFSI in EC both systems are charged but the charge increases from −*e* in the oxidized form to −2 *e* in the reduced form.

In the vacuum calculations, the trends were different. Although only in some cases was it possible to extrapolate the data to infinite *N*, it is clear from Figure 1 than the difference between −EA and *E*_LUMO_ remains fairly constant for all system sizes. The relative changes for different types of the system are opposite to those observed in PCM: presence of Li^+^ lowers the energy and the energies are increased for systems containing TFSI anion.

The approximation that −EA ≅ *E*_LUMO_ assumes that the orbitals and their energies do not change significantly when an electron is added to the system. Seeking for some clues about the origin of different behavior of the vacuum and the PCM results, we examined the orbitals and electron densities of selected EC and LiTFSI/EC systems. We chose 10–15 structures for which the *E*_LUMO_ was close either to the average or to the extreme values and visually inspected frontier orbitals (HOMO and LUMO) of the closed-shell system and the singly occupied molecular orbital (SOMO) of the open-shell system. We also plotted the difference Δρ between the electron densities of open-shell and closed-shell system, that is, the change of the density when the additional electron is added to the cluster.

**Figure 5:**
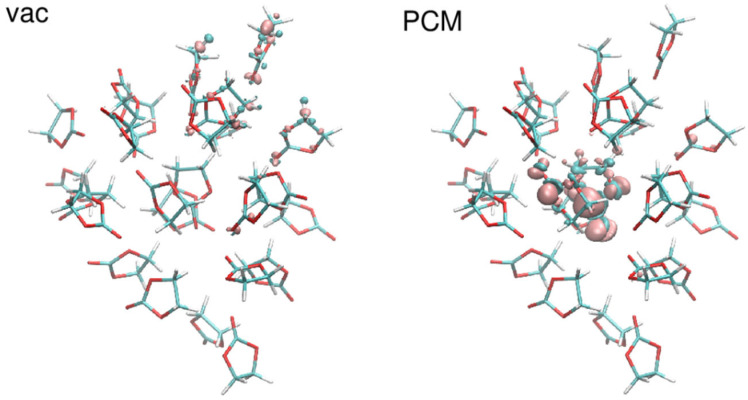
Difference in the electron density between reduced and oxidized state of a system of 25 EC molecules. Positive values—cyan; negative values—magenta.

For the EC aggregates, the main difference between vacuum and PCM results is that in vacuum the LUMO of the neutral system (and accordingly SOMO of the system with negative charge) is located at the surface of the cluster, and spreads over several molecules; such spread is often larger for SOMO. In PCM there is also a tendency of LUMO (SOMO) location at the surface, but more often LUMO and SOMO are contained to a single molecule. There are also some cases in which LUMO is located inside the aggregate. Accordingly, in vacuum the density difference Δρ between charged and neutral system is located at the surface and dispersed over many molecules, whereas it is more localized in PCM calculations. In Figure 5 we show an extreme example of an aggregate for which Δρ is located in the center of the cluster in PCM calculations but in vacuum it spreads over four molecules at the surface.

**Figure 6:**
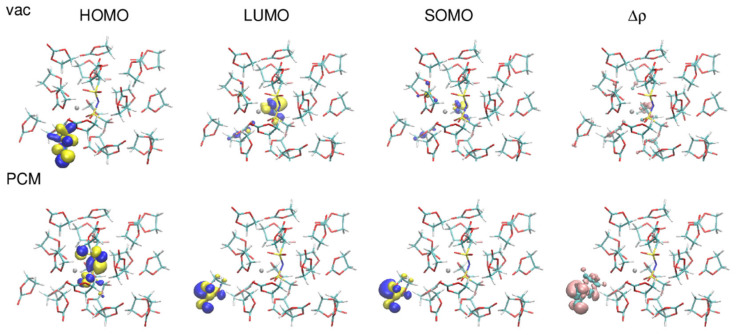
Plots of HOMO, LUMO, SOMO orbitals and the difference in the electron density between reduced and oxidized state obtained for a Li-TFSI ion pair solvated in 20 EC molecules.

Even larger differences between orbitals calculated in vacuum and in the implicit solvent are noticeable for LiTFSI/EC systems. A typical example is presented in Figure 6. In PCM calculations HOMO resides on the TFSI anion, in vacuum it is usually located on an EC molecule at the surface. SOMO in PCM is more localized than in vacuum, therefore also the density difference Δρ obtained in PCM is contained to fewer (often to solely one) molecules than in the vacuum results. It can be noted, that in PCM Δρ is localized on the same molecule as LUMO, whereas it is more dispersed in vacuum.

We concluded therefore, that the origin of the different trends exhibited by vacuum and PCM results stems from the different behavior of frontier orbitals. In PCM LUMO ≈ SOMO (and Δρ), therefore the same molecules are important in calculations of −EA and *E*_LUMO_, thus for large systems values of these two parameters converge. In vacuum, different parts of the system may be involved in LUMO and SOMO, so that one cannot expect that LUMO energy will be an accurate approximation of electron affinity. Briefly, the ineffectiveness of the explicit solvent model can be explained by the fact that the important orbitals tend to be located at the surface of the aggregate, so that they are exposed to vacuum, regardless of the system size. In the hybrid model, the system is embedded in a polarizable continuum, yielding stabilization to the surface states.

We should note that the solvent effect on the HOMO and LUMO energies is relevant not only to the redox potentials and the electrochemical windows [18,19] but also to the modeling of other properties related to HOMO-LUMO gap, as the energies of optical transitions [20,21,22] or charge transport in semiconductors [23]. Therefore, our findings may be useful e.g., for computational modeling of absorption spectra in solutions.

The PCM *E*_LUMO_ (or equivalently −EA) value listed in Table 2 may be used to estimate the reduction potential of EC. The correction of 1.39 eV is subtracted from the reduction potential to convert it to the scale relative to Li^+^|Li electrode [24]. This procedure yields 0.5 eV as the reduction potential of EC. The value is lower than the experimental result 1.36 eV [25], but it is to be expected, as proper reproduction of the potential requires bond cleavage in the reduced state [2]. This has not been taken into account in our calculations aimed at the investigations of the solvent model.

Differences between the PCM *E*_LUMO_ values for neat solvent aggregates and those containing ions suggested that the presence of dissolved salt affects the reduction potential of the solvent and should be accounted for in the modeling of redox potentials of the electrolyte. The disadvantage of the hybrid model used in our calculations is that it contains a boundary between explicit and continuous solvent, which may lead to artefacts caused by surface states. The more realistic approach (yet computationally expensive) to the modeling of redox potentials of liquids and electrolyte solutions seems therefore the use of ab initio molecular dynamics to model the liquid in periodic boundary conditions. In this way the surface of the system is eliminated, and the effects of dissolved salt can be easily modeled by changing the concentration. Calculations of redox potentials for entire liquid structure in a periodic box were performed e.g., for ionic liquids [18]. We plan such investigations on salt solutions in EC as the subject of a future study.

## 4. Materials and Methods

In this work we conducted quantum-chemical calculations for a series of clusters of EC molecules. Increasing size of these clusters accounted therefore for solvation effects in an explicit solvent model. The hybrid (explicit/continuous) solvent model was tested by embedding the clusters in an implicit continuous solvent, providing the correction from the dielectric continuum surrounding the explicit part of the system. We also performed a series of calculations for EC clusters containing salt ions (Li^+^ and/or TFSI), as the presence of dissolved ions can affect the calculated reduction potential of the solvent [26].

Structures of molecular aggregates of EC were obtained from the trajectories recorded for neat solvent during classical MD simulations of EC-based electrolytes reported in our recent study [27]. The aggregate was constructed by randomly choosing an EC molecule from a frame of the MD trajectory and then selecting the desired number of EC molecules closest to it. The number *N* of EC molecules in the aggregate varied between 1 and 25. This procedure was repeated for 100 frames, spanning the time of 20 ns in the MD simulations. In this way we prepared sets of data for *N* = 1, 2, 5, 10, 15, 20 and 25; each set consisted of 100 structures.

Sets of structures of an ion or Li-TFSI ion pair solvated in EC were constructed in similar manner. In this case we wanted to avoid including multiple ions (or ion pairs) in the cluster, therefore we performed additional MD runs for a system of a single LiTFSI pair in a box of 1500 EC molecules using the same force field and simulation conditions as in the work [27]. Then, the charged structures of central ion with solvated molecules were extracted from the frames with a large separation between anion and cation, whereas the aggregates with solvated ion pair were obtained from the frames where the distance between the Li^+^ ion and the nearest oxygen atom from the TFSI anion was not larger than 2.6 Å. The sets of 100 structures of solvated TFSI anion or Li-TFSI pair were prepared for *N* = 1, 2, 5, 10, 15 and 20 EC molecules and the 100 structures with Li^+^ cation contained *N* = 1, 2, 4, 10, 15 or 20 solvent molecules. The value *N* = 4 was used because four EC molecules complete the solvation shell of the cation.

Single-point energy calculations were performed using Gaussian 09 rev. D.01 [28]. The DFT methodology with B3LYP functional and the 6-31++G** basis set was used. Two types of calculations were performed for each structure: closed shell calculations (with total charge equal 0 for EC aggregates or for solvated Li-TFSI pair, charge +1 for solvated cation and with charge −1 for solvated anion), and open-shell calculations for structures with an additional electron (with charges −1, 0 and −2 for EC aggregates or solvated LiTFSI, solvated Li^+^, and solvated TFSI, respectively).

Each computation was performed in a vacuum and then repeated for the system embedded in the effective solvent modeled via polarizable continuum model with the static dielectric constant set to the value 90.5, corresponding to EC liquid. The default scaled van der Waals surface was used for the cavity construction. For *N* > 1, the PCM calculations constituted, therefore, a hybrid solvent model in which part of the solvent was treated explicitly as *N* solvent molecules and the effect of the dielectric continuum was included through the continuous PCM solvent.

Electron affinity of each system was computed as the energy difference between the system with an additional electron and the system with the original charge:

EA = −(*E*_O_ − *E*_C_) = *E*_C_ − *E*_O_, (9)

where *E*_O_ and *E*_C_ are the energies obtained in the open-shell and closed-shell calculations, respectively. Energies of the lowest virtual orbital *E*_LUMO_ and the energies of the highest occupied molecular orbital *E*_HOMO_ were extracted from the closed-shell results.

In addition to the single-point energy calculations for clusters, we also performed vacuum and PCM geometry optimizations and harmonic frequency calculations for a single EC molecule and an EC anion (no ring opening was modeled in the anion), to obtain some data for comparison.

## Figures and Tables

**Table 1:** Gibbs free energy change in the EC reduction, electron affinity and the LUMO energy of EC molecule calculated at the B3LYP/6-31++G** level in vacuum and in the PCM solvent.

Energy	Vacuum	PCM
–ΔG (eV)	0.26	−1.62
−EA (eV)	0.31	−1.48
*E*_LUMO_ (eV)	−0.61	−0.21

**Table 2:** Electron affinity and LUMO energy calculated in vacuum and in the PCM for aggregates of EC molecules or solvated ions, extrapolated to an infinite system size. All values in eV.

Core	*E*_LUMO_ (vac)	−EA (vac)	*E*_LUMO_ (PCM)	−EA (PCM)
EC	−3.38	−2.49	−1.94	−1.90
Li^+^			−1.57	−1.56
TFSI	−3.16	−1.90	−2.03	−2.07
Li-TFSI			−1.73	−1.76

**Table 3:** HOMO–LUMO gap obtained from the extrapolation to an infinite system size for aggregates of EC molecules or solvated ions. All values in eV.

Core	Δ_H-L_ (Vacuum)	Δ_H-L_ (PCM)
EC	2.59	5.91
Li^+^		5.84
TFSI		5.89
Li-TFSI		6.39

## Data Availability

Raw data are available upon a request to the corresponding author.
